# Methodologies to measure the coverage of vitamin A supplementation: a systematic review

**DOI:** 10.1017/jns.2021.65

**Published:** 2021-08-27

**Authors:** Alessandro Miglietta, Annette Imohe, Andreas Hasman

**Affiliations:** 1 Independent Consultant, Nutrition Section, UNICEF Headquarters, New York, USA; 2 Nutrition Section, UNICEF Headquarters, New York, USA

**Keywords:** Epidemiologic methods, Epidemiologic monitoring, Systematic review, Vitamin A, CCS, coverage cluster survey, CLQAS, clustered lot quality assurance sampling, EPI, Expanded Programme on Immunization, HH, household, VAD, vitamin A deficiency, VAS, vitamin A supplementation, WHO, World Health Organization

## Abstract

Countries are increasingly transitioning from event-based vitamin A supplementation (VAS) distribution to delivery through routine health system contacts, shifting also to administrative, electronic-based monitoring tools, a process that brings certain limitations affecting the quality of administrative VAS coverage. At present, there is no standardised methodology for measuring the coverage of VAS delivered through routine health services. To address this gap, we conducted a systematic review of the literature to identify and recommend methods to measure VAS coverage, with the aim of providing guidance to countries on the collection of consistent data for planning, monitoring and evaluating VAS programmes integrated into routine health systems. We searched the PubMed®, Embase®, Scopus, Google Scholar and World Health Organization (WHO) Global Index Medicus databases for studies published from 1 January 2000 to 1 January 2021, reporting original data on VAS coverage and methodologies used for measurement. We screened 2371 original titles and abstracts, assessed twenty-seven full-text articles and ultimately included eighteen studies. All but two studies used a coverage cluster survey (CCS) design to measure VAS coverage, adapting the WHO Vaccination Coverage Cluster Surveys methodology, by modifying sample size and sampling parameters. Annual two-dose VAS coverage was reported from only four studies. Until electronic-based systems to collect and analyse VAS data are equipped to measure routine two-dose VAS coverage using administrative data, CCSs that comply with the 2018 WHO Vaccination Coverage Cluster Surveys Reference Manual represent the gold-standard method for effective VAS programme monitoring.

## Introduction

Vitamin A supplementation (VAS) is a highly cost-effective public health intervention that reaches approximately 250 million children every year, protecting them from blindness and decreasing their risk of mortality from preventable causes^(1)^. In settings where vitamin A deficiency (VAD) is a public health problem, the World Health Organization (WHO) recommends two high-dose vitamin A supplements annually, spaced 4–6 months apart, for children aged 6–59 months^(2,3)^. UNICEF estimates that VAD affected about one-third of children aged 6–59 months in 2018, with the highest rates in sub-Saharan Africa (48 %) and South Asia (44 %)^(4)^.

Since 1998, VAS has been delivered mainly through campaigns, such as polio supplementary immunization activities (SIAs)^(3)^. Recently, the high cost of campaign delivery and the reduced frequency and geographical distribution of polio-SIAs due to effective eradication efforts, have encouraged countries to increasingly use routine health services for VAS delivery^(4)^. This change in delivery mechanism has coincided with a shift in programme monitoring tools, with many countries moving from paper-based to electronic-based administrative monitoring systems^(4)^.

The main indicator for VAS programme monitoring is VAS coverage, defined as the percentage of children aged 6–59 months of age receiving an age-appropriate vitamin A supplement in each of two annual semesters^(5,6)^.

In settings where VAS is distributed through campaigns, there is existing guidance on how to measure and validate VAS coverage after an event using survey methods, including the Post Event Coverage Survey (PECS)^(7)^, which employs the Expanded Programme on Immunization (EPI) cluster survey methodology^(8)^. However, this technique was revised by WHO in response to methodological concerns^(9)^. The main changes brought by the 2018 WHO Vaccination Coverage Cluster Surveys Reference Manual include the use of probability-based sampling methods at each stage; households (HHs) selected by a central group of planners rather than interviewers in the field; interview of every eligible child in the HH; and weighted analysis.

Other tools used to measure VAS coverage are represented by large-scale multi-topic HH surveys, such as the Demographic and Health Survey^(10)^ or the UNICEF Multiple Indicator Cluster Survey^(11)^. However, such HH surveys have limitations in supporting VAS programme management needs, as they are not designed to measure annual two-dose VAS coverage; moreover, they are expensive and carried out too infrequently (i.e. every 10 years) to allow a real-time monitoring of VAS coverage aimed to identify and implement corrective actions (i.e. supplementary VAS activities in specific areas).

At present, there is no standardised methodology for measuring and validating the coverage of VAS delivered through routine health services. Strengthened methods are therefore required for accurate and timely measurement of VAS coverage. This is particularly important as countries integrate VAS into the routine health systems and shift to administrative, electronic-based monitoring tools, a process that brings certain limitations affecting the quality of administrative VAS coverage, which can impair effective VAS programme monitoring^(5,6)^.

To address this gap, we conducted a systematic review of the literature to identify and recommend methods to measure VAS coverage, with the aim of providing guidance to countries on the collection of consistent data for planning, monitoring and evaluating VAS programmes integrated into routine health systems.

## Methods

The review followed the Preferred Reporting Items for Systematic Reviews and Meta-Analyses (PRISMA) guidelines^(12)^. The protocol for the review was not registered on the PROSPERO register of systematic reviews but is available on request.

We searched the PubMed®, Embase®, Scopus, Google Scholar and WHO Global Index Medicus databases for peer-reviewed studies reporting original data on VAS coverage among children under 5 years of age and methodologies used for measurement.

To focus on methods currently in use, we only included articles published from 1 January 2000 to 1 January 2021. Studies written in languages other than English, French, Portuguese or Spanish were excluded.

We used a combination of medical subject headings (MeSH) and text words, Boolean operators and synonyms in the thesaurus to create database-appropriate syntax (Table 1).

**Table 1.** PubMed® search strategy used in the systematic review of methodologies to measure Vitamin A Supplementation Coverage

Vitamins[MeSH] OR vitamin A[MeSH] OR Vitamin A Deficiency[MeSH] OR micronutrien[MeSH] OR nutrients[MeSH] OR dietary supplements[MeSH] OR Capsules[MeSH] OR immunization[MeSH] OR vaccination[MeSH] OR vaccines[MeSH] OR “immunization programs”[MeSH] OR “parasitic diseases”[MesH] OR vitamin*[Title/Abstract] OR micronutrien*[Title/Abstract] OR nutrient*[Title/Abstract] OR immunization[Title/Abstract] OR immunisation[Title/Abstract] OR vaccination[Title/Abstract] OR vaccin*[Title/Abstract] OR deworm*[Title/Abstract] OR schistosom*[Title/Abstract] OR filariasis*[Title/Abstract] OR parasitic*[Title/Abstract] OR trachoma*[Title/Abstract] OR onchocercia*[Title/Abstract] OR malaria*[Title/Abstract] AND coverage[Title/Abstract] OR validat*[Title/Abstract] OR compar*[Title/Abstract] OR assess*[Title/Abstract] OR evaluat*[Title/Abstract] OR estimat*[Title/Abstract] OR measur*[Title/Abstract]

*Note*: Search strategies for other databases used (Embase®, Scopus, Google Scholar and WHO Global Index Medicus) are available from the corresponding author.

As vitamin A supplement is delivered together with other health interventions, as immunisations, deworming and other parasite control programmes, the search strategy also includes such-related terms.

Bibliographic information was imported into a citation bibliographic management software for the storage and removal of duplicates. After duplicate citations were removed, titles and abstracts were independently screened for eligibility by authors. The reference lists of relevant articles were also checked to identify further eligible studies. In cases of disagreement, consensus was sought after reading the full-text article.

As all the studies included in the systematic review adopted a cross-sectional design, quality was assessed by two authors using the Newcastle–Ottawa Scale adapted for cross-sectional studies^(13)^. Disagreements in quality assessment were resolved through discussion.

Authors extracted the data using an electronic form. The summary of findings tables accompanied by a narrative synthesis was used to synthetise and present results.

The data collected included bibliographic information (authors, year and country of publication); study design; sample size and sampling procedures; data collection methods; data quality assurance methods; data analysis methods; ethical considerations; planning considerations (e.g. number of personnel, study length and month of study implementation) and outcome measured (routine/after event one-dose and/or two-dose VAS coverage). The terminology used in the systematic review is provided in the glossary of terms (Supplementary material 1).

## Results

**Fig. 1.**
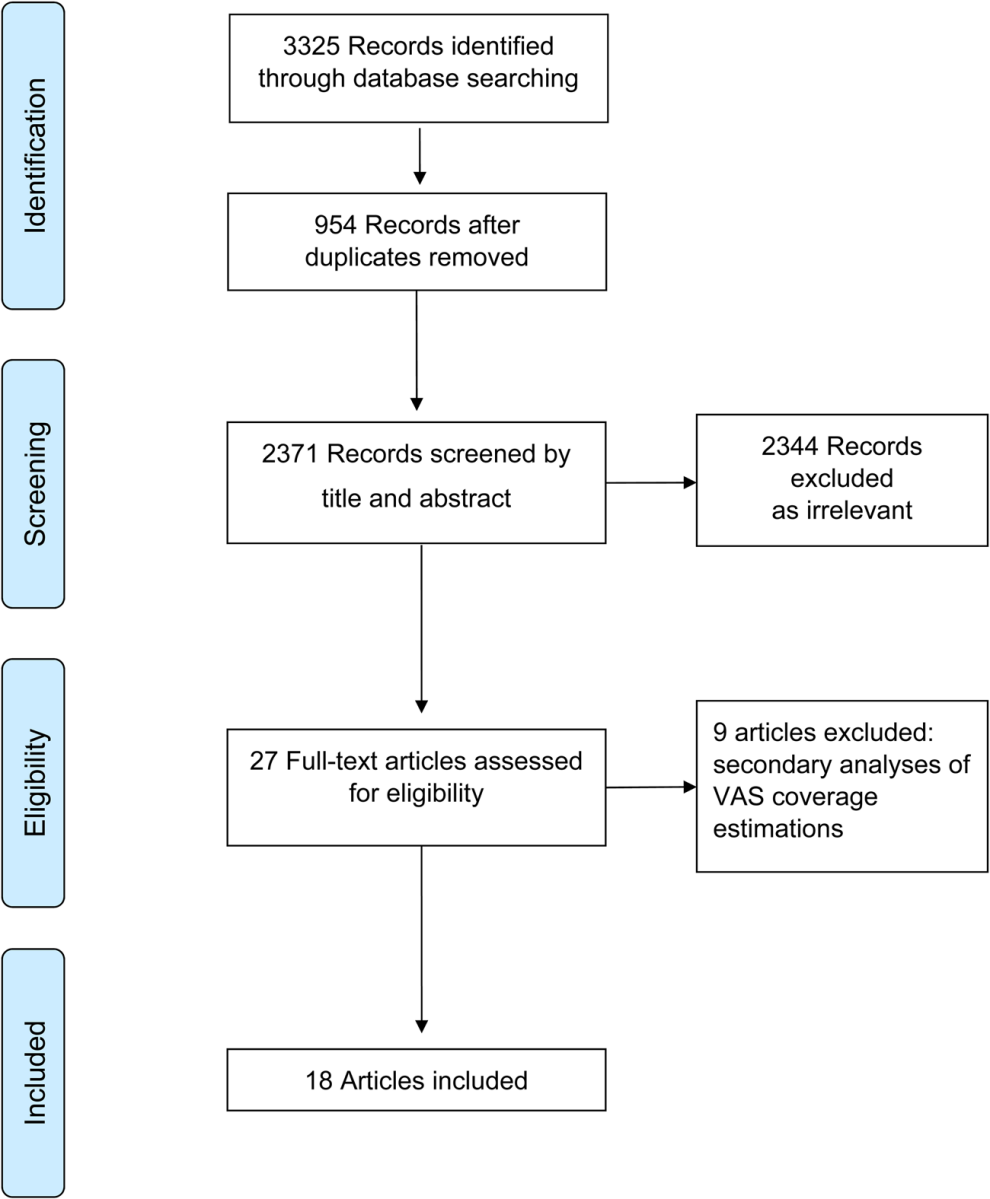
Flowchart of the selection of studies included in the systematic review of methodologies to measure the coverage of vitamin A supplementation (VAS).

We identified 3325 abstracts through database searches. After removing duplicates and screening out non-relevant abstracts, we assessed twenty-seven full-text articles for eligibility. Of these, eighteen studies that met the selection criteria were included in the systematic review (Fig. 1)^(14–31)^.

Fifteen studies were conducted in the WHO Africa Region^(14–17,19–25,27–31)^ and three in the WHO South-East Asia Region^(15,18,26)^ during the period 2001–2020. When appraised for quality, only five studies were categorised as high quality^(19,21,25,29,30)^ (Table 2).

**Table 2.** Quality appraisal of the eighteen studies included in the systematic review of methodologies to measure vitamin A supplementation coverage – Newcastle–Ottawa Scale (adapted for cross-sectional studies)^(11)^

Study	Selection				Comparability	Outcome/Exposure		Total stars	Quality rating
	Sample representative	Sample size	Non-respondents	Ascertainment of exposure	Control of confounding factos	Assessment of outcome	Statistical test		
Bharmal (2001), Pakistan^(14)^				*		*		2	Low
Masanja (2006), Tanzania^(15)^	*	*		*	**	*	*	7	Medium
Bendech (2007), Guinea^(16)^	*			*	**	*	*	6	Medium
Ayoya (2007), Mali^(17)^	*			*	*	*	*	5	Medium
Sachdeva (2009), India^(18)^	*			*		*		3	Low
Gebremedhin (2009), Ethiopia^(19)^	*	*	*	**	*	*	*	8	High
Hodges (2013), Sierra Leone^(20)^	*			**	*	*		5	Medium
Nyhus (2013), Tanzania^(21)^	*	*		**	**	*	*	8	High
Olusegun (2013), Zambia^(22)^	*	*		**	*	*	*	7	Medium
Hamadoun (2013), Mali^(23)^	*	*		*		*	*	5	Medium
Clohossey (2014), Kenya^(24)^	*			*		*	*	4	Low
Sesay (2015) Sierra Leone^(25)^	*	*	*	**	*	*	*	8	High
Harsh (2015), India^(26)^	*	*	*	*	*	*	*	7	Medium
Ouédraogo (2016), Burkina Faso^(27)^	*			*	*	*	*	5	Medium
Doris (2016), Ghana^(28)^	*	*		*	*	*		5	Medium
Adamu (2016), Nigeria^(29)^	*	*	*	**	*	*	*	8	High
Koroma (2020), Sierra Leone^(30)^	*	*	*	**	*	*	*	8	High
Kassa (2020), Ethiopia^(31)^	*	*		*	*	*	*	6	Medium

Quality threshold: high quality: eight to ten stars; medium quality: five to seven stars; low quality: zero to four stars.

Newcastle–Ottawa Quality Assessment Scale (adapted for cross-sectional studies)

Selection: (Maximum five stars)

(1) Representativeness of the sample:

(a) Truly representative of the average in the target population. * (all subjects or random sampling)

(b) Somewhat representative of the average in the target population. * (non-random sampling)

(c) Selected group of users.

(d) No description of the sampling strategy.

(2) Sample size:

(a) Justified and satisfactory. *

(b) Not justified.

(3) Non-respondents:

(a) Comparability between respondents and non-respondents characteristics is established, and the response rate is satisfactory. *

(b) The response rate is unsatisfactory, or the comparability between respondents and non-respondents is unsatisfactory.

(c) No description of the response rate or the characteristics of the responders and the non-responders.

(4) Ascertainment of the exposure (risk factor):

(a) Validated measurement tool. **

(b) Non-validated measurement tool, but the tool is available or described.*

(c) No description of the measurement tool.

Comparability: (Maximum two stars)

(1) The subjects in different outcome groups are comparable, based on the study design or analysis. Confounding factors are controlled.

(a) The study controls for the most important factor (select one). *

(b) The study control for any additional factor. *

Outcome: (Maximum three stars)

(1) Assessment of the outcome:

(a) Independent blind assessment. **

(b) Record linkage. **

(c) Self-report. *

(d) No description.

(2) Statistical test:

(a) The statistical test used to analyse the data is clearly described and appropriate, and the measurement of the association is presented, including confidence intervals and the probability level (*P* value). *

(b) The statistical test is not appropriate, not described or incomplete.

Sixteen studies employed a coverage cluster survey (CCS) design^(14–26,28,29,31)^, one used a Longitudinal Cluster Survey (LCS) methodology^(27)^ and one a clustered lot quality assurance sampling (CLQAS) approach^(30)^. Half of the studies (*n* 9) were conducted at the country level^(15–17,20–25)^ and the other half were conducted at the subnational level (e.g. city, district or region). The majority of articles included (*n* 16) enrolled the whole VAS target age group of children aged 6–59 months^(14–17,19–21,23–31)^.

### Sample size and sampling procedures

Overall, six studies^(14,16,18,20,24,27)^ did not report procedures employed to calculate the sample size (Table 3).

**Table 3.** Sample size procedures and characteristics of studies included in the systematic review of methodologies to measure the coverage of VAS (*n* 18)

Article	Study design	Setting	Population	Sample size dimension (*n*)	Anticipated VAS coverage considered	Desired precision	Confidence level	Non-response rate	Design effect
Bharmal (2001), Pakistan^(14)^	CCS	Municipality	Children 6–59 months	443	NR	NR	NR	NR	NR
Masanja (2006), Tanzania^(15)^	CCS	Country	Children 6–59 months	2400	Yes	10 %	95 %	NR	NR
Bendech (2007), Guinea^(16)^	CCS	Country	Children 6–59 months	1950	NR	NR	NR	NR	NR
Ayoya (2007), Mali^(17)^	CCS	Country	Children 6–59 months	210	NR	NR	95 %	NR	NR
Sachdeva (2009), India^(18)^	CCS	Municipality	Children 12–59 months	210	NR	NR	NR	NR	NR
Gebremedhin (2009), Ethiopia^(19)^	CCS	District	Children 6–59 months	2400	Yes	5 %	95 %	NR	NR
Hodges (2013), Sierra Leone^(20)^	CCS	Country	Children 6–59 months	900	NR	NR	NR	NR	NR
Nyhus (2013), Tanzania^(21)^	CCS	Country	Children 6–59 months	1200	NR	5 %	95 %	NR	NR
Olusegun (2013), Zambia^(22)^	CCS	Country	Children 12–59 months	360	Yes	5 %	95 %	NR	2⋅0
Hamadoun (2013), Mali^(23)^	CCS	Country	Children 6–59 months	1700	Yes	5 %	95 %	NR	2⋅0
Clohossey (2014), Kenya^(24)^	CCS	Country	Children 6–59 months	900	NR	NR	NR	NR	NR
Sesay (2015), Sierra Leone^(25)^	CCS	Country	Children 6–59 months	5880	Yes	5 %	95 %	NR	NR
Harsh (2015), India^(26)^	CCS	Municipality	Children 6–59 months	210	Yes	5 %	95 %	5 %	2⋅0
Ouédraogo (2016), Burkina Faso^(27)^	LCS	District	Children 6–59 months	10 454	NR	NR	NR	NR	NR
Doris (2016), Ghana^(28)^	CCS	District	Children 6–59 months	418	Yes	6 %	95 %	5 %	1⋅5
Adamu (2016), Nigeria^(29)^	CCS	District	Children 6–59 months	900	Yes	5 %	95 %	10 %	5⋅0
Koroma (2020), Sierra Leone^(30)^	CLQAS	District	Children 6–59 months	855	NR	10 %	95 %	NR	NR
Kassa (2020), Ethiopia^(31)^	CCS	District	Children 6–59 months	840	Yes	5 %	95 %	10 %	2⋅0

CCS, coverage cluster survey; LCS, longitudinal cluster survey; CLQAS, clustered lot quality assurance sampling survey; NR, not reported; VAS, vitamin A supplementation.

Anticipated VAS coverage was considered by nine studies^(15,19,22,23,25,28,29,31)^, which together with other two studies^(21,30)^ defined a desired precision ranging between ±5 and 10 %. A confidence level of 95 % was set by twelve studies^(15,17,19,21–23,25,26,28–31)^.

Six articles^(22,23,26,28,29,31)^ considered the design effect (DEFF) in the sample size calculation process, using a value between 1⋅5 and 5⋅0 (mean 2⋅4; median 2⋅0).

Only four studies^(26,28,29,31)^ further increased the sample size for an estimated non-response rate, which ranged between 5 and 10 % (mean and median 7⋅5 %).

There was no relation between the sample dimension, the geographical level where studies were conducted and their design: CCs reported a sample size ranging between 210 units at the municipality level^(17)^ to 5880 units at the national level^(25)^, the CLQAS^(30)^ and LCS^(27)^ sampled 855 and 10 454 units at the district level, respectively.

Concerning sampling procedures (Table 4), all the included studies used a multistage cluster sampling design. At the first stage, the majority of studies (*n* 11)^(14,15,17–19,26–31)^ selected a subnational administrative division (i.e. districts, provinces and regions) by convenience, and within such strata, most (*n* 15)^(15,16,18–26,28–31)^ sampled clusters with probability proportional to their size (PPS). Seven studies^(16,19,20,22,23,25,31)^ defined clusters as enumeration areas (EAs) and five^(21,22,25,30)^ used the segmentation technique for cluster selection.

**Table 4.** Sampling procedures used by studies included in the systematic review of methodologies to measure the coverage of vitamin A supplementation (*n* 18)

Article	Setting	Strata selection and definition	Cluster definition	Cluster selection	Cluster segmentation	Number of clusters	Number of HHs	HH selection	Child selection
Bharmal (2001), Pakistan^(14)^	Municipality	Convenience selection of 1 municipality	Block	Convenience selection	No	1	All HHs visited	Convenience selection	One per HH
Masanja (2006), Tanzania^(15)^	Country	Convenience selection of 4 districts	Health zones	PPS	No	30 per strata	20 per cluster	WHO random walk method	One per HH
Bendech (2007), Guinea^(16)^	Country	Country divided into 5 zones (rural/urbans) and all selected	Enumeration areas	PPS	No	30 per strata	15 per cluster	WHO random walk method	One per HH
Ayoya (2007), Mali^(17)^	Country	Convenience selection of 7 regions	Municipality	Random	No	1 per strata	30 per cluster	WHO random walk method	One per HH
Sachdeva (2009), India^(18)^	Municipality	Convenience selection of 1 municipality	Slums	PPS	No	30	7 per cluster	WHO random walk method	One per HH
Gebremedhin (2009), Ethiopia^(19)^	District	Convenience selection of 1 district	Enumeration areas	PPS	No	80	30 per cluster	WHO random walk method	One per HH
Hodges (2013), Sierra Leone^(20)^	Country	30 EAs selected at country level with PPS	Enumeration areas	PPS	No	30	30 per cluster	WHO random walk method	One per HH
Nyhus (2013) Tanzania^(21)^	Country	30 villages selected at country level with PPS	Villages	PPS	Yes	30	40 per cluster	WHO random walk method	One per HH
Olusegun (2013) Zambia^(22)^	Country	40 EAs selected at country level with PPS	Enumeration areas	PPS	Yes	40	9 per cluster	WHO random walk method	All eligible within the HH
Hamadoun (2013), Mali^(23)^	Country	Random selection of 4 districts	Health Centers	PPS	No	25 per strata	17 per cluster	WHO random walk method	All eligible within the HH
Clohossey (2014), Kenya^(24)^	Country	30 EAs selected at the country level with PPS	Enumeration areas	PPS	Yes	30	30 per cluster	WHO random walk method	One per HH
Sesay (2015), Sierra Leone^(25)^	Country	All the 14 countries’ districts selected	Enumeration areas	PPS	Yes	30 per strata	14 per cluster	HHs randomly selected from a full-list	One per HH
Harsh (2015), India^(26)^	Municipality	Convenience selection of 1 municipality	Lanes	PPS	No	30	7 per cluster	WHO random walk method	One per HH
Ouédraogo (2016), Burkina Faso^(27)^	District	Convenience selection of 1 district	Health Centers	Convenience selection	No	24	All HHs visited	Convenience selection	All eligible within the HH
Doris (2016), Ghana^(28)^	District	Convenience selection of 1 district	Communities	PPS	No	4	All HHs visited	Convenience selection	All eligible within the HH
Adamu (2016), Nigeria^(29)^	District	Convenience selection of 1 district	Villages	PPS	No	20	45 per cluster	WHO random walk method	All eligible within the HH
Koroma (2020), Sierra Leone^(30)^	District	Convenience selection of 3 districts	Villages	PPS	Yes	19 per strata	19 per cluster	HHs randomly selected from a full-list	One per HH
Kassa (2020), Ethiopia^(31)^	District	Convenience selection of 1 district	Enumeration areas	PPS	No	39	NA	NA	Children randomly selected from a full list

CCS, coverage cluster survey; EAs, census enumeration areas; HHs, households; LCS, longitudinal cluster survey; NA, not applicable; NR, not reported; PPS, probability proportional to size; WHO, World Health Organization.

Within the selected clusters, all but one study^(31)^ sampled HHs to find eligible children, with the majority (*n* 12)^(15–24,26,29)^ using the WHO random walk method. Five articles^(22,23,27–29)^ reported that all eligible children within a selected HH were enrolled, while the other thirteen studies reported selecting only one eligible child by random selection. In all the reviewed studies, parents or caregivers were interviewed to collect information about VAS received.

Overall, the number of selected clusters per strata ranged from four to eighty, with eight studies^(15,16,18,20,21,24–26)^ opting for thirty clusters. The number of sampled HHs per cluster ranged from seven to forty-five (mean 22⋅5; median 20).

### Data collection and quality assurance

The data collection and quality assurance procedures used in the reviewed studies are presented in Table 5. Only two studies^(28,30)^ used an electronic tool to collect information on the number of vitamin A supplements received by enrolled children, while the other studies used a standardised questionnaire, which was pre-tested and translated in the local language in thirteen studies^(14,16,19–25,27,29–31)^.

**Table 5.** Data collection and quality assurance procedures adopted by studies included in the systematic review of methodologies to measure the coverage of vitamin A supplementation (*n* 18)

Article	Data collection tool	Evidence of data collection	Data collection tool pre-tested and translated	Samples of vitamin A supplementation capsules shown to parents	Training provided	Interviewers supervised	Revisit/replace plan for clusters and HHs	Double data entry and cleaning performed
Bharmal (2001), Pakistan^(14)^	Questionnaire	By card plus recall	Yes	No	No	Yes	No	Yes
Masanja (2006), Tanzania^(15)^	Questionnaire	By card plus recall	No	Yes	Yes	No	No	Yes
Bendech (2007), Guinea^(16)^	Questionnaire	By card plus recall	Yes	Yes	Yes	Yes	Yes	Yes
Ayoya (2007) Mali^(17)^	Questionnaire	By card plus recall	No	Yes	Yes	No	No	No
Sachdeva (2009), India^(18)^	Questionnaire	By card plus recall	No	No	No	No	No	No
Gebremedhin (2009), Ethiopia^(19)^	Questionnaire	By card plus recall	Yes	Yes	Yes	Yes	No	Yes
Hodges (2013), Sierra Leone^(20)^	Questionnaire	By card plus recall	Yes	Yes	Yes	No	No	Yes
Nyhus (2013), Tanzania^(21)^	Questionnaire	By card plus recall	Yes	Yes	No	Yes	No	Yes
Olusegun (2013), Zambia^(22)^	Questionnaire	By card plus recall	Yes	Yes	No	No	No	Yes
Hamadoun (2013), Mali^(23)^	Questionnaire	By card plus recall	Yes	No	No	Yes	No	No
Clohossey (2014), Kenya^(24)^	Questionnaire	By card plus recall	Yes	Yes	No	Yes	No	Yes
Sesay (2015), Sierra Leone^(25)^	Questionnaire	By card only	Yes	No	Yes	Yes	No	Yes
Harsh (2015), India^(26)^	Questionnaire	By card plus recall	No	No	No	No	No	No
Ouédraogo (2016), Burkina Faso^(27)^	Questionnaire	By card plus recall	Yes	Yes	Yes	Yes	No	Yes
Doris (2016), Ghana^(28)^	Electronic toll	By card plus recall	No	No	No	No	No	No
Adamu (2016), Nigeria^(29)^	Questionnaire	By card plus recall	Yes	No	No	No	No	Yes
Koroma (2020), Sierra Leone^(30)^	Electronic toll	By card plus recall	Yes	Yes	Yes	Yes	No	No
Kassa (2020), Ethiopia^(31)^	Questionnaire	By card plus recall	Yes	Yes	Yes	No	No	Yes

HHs, households.

As evidence of VAS, all the included studies collected information using parental recall when the child health card (CHC) was not available. In addition, a sample of VAS capsules was shown to parents to reduce recall biases in eleven articles^(15–17,19–22,24,27,30,31)^.

Ten studies^(14–17,19,20,25,27,30,31)^ provided 2 to 5 days of training to interviewers, and nine conducted field supervision^(14,16,19,21,23–25,27,30)^.

The data collected were double-checked and cleaned for analysis in twelve studies^(14–16,19–22,24,25,27,29,31)^. Only one study^(16)^ reported having a plan to replace or revisit empty HHs and clusters that had suddenly become inaccessible.

### Ethical and planning consideration

Verbal consent was requested from parents of eligible children in fifteen studies^(14–16,19–24,26–31)^ and twelve studies^(15,16,19–21,23,26–31)^ also obtained ethical clearance from an ethics committee (Table 6).

**Table 6.** Planning and ethical considerations of studies included in the systematic review of methodologies to measure the coverage of vitamin A supplementation (*n* 18)

Article	Number of interviewers	Number of supervisors	Study length	Ethical approval obtained	Parental verbal informed consent requested
Bharmal (2001), Pakistan^(14)^	72	7	NR	No	Yes
Masanja (2006), Tanzania^(15)^	NR	NR	NR	Yes	Yes
Bendech (2007), Guinea^(16)^	30	10	NR	Yes	Yes
Ayoya (2007), Mali^(17)^	NR	NR	NR	NR	NR
Sachdeva (2009), India^(18)^	NR	NR	NR	NR	NR
Gebremedhin (2009), Ethiopia^(19)^	18	6	4 weeks	Yes	Yes
Hodges (2013), Sierra Leone^(20)^	10	NR	1 week	Yes	Yes
Nyhus (2013), Tanzania^(21)^	36	3	6 weeks	Yes	Yes
Olusegun (2013), Zambia^(22)^	NR	NR	NR	No	Yes
Hamadoun (2013), Mali^(23)^	NR	NR	2 weeks	Yes	Yes
Clohossey (2014), Kenya^(24)^	15	5	4 weeks	No	Yes
Sesay (2015), Sierra Leone^(25)^	53	13	5 d	NR	NR
Harsh (2015), India^(26)^	NR	NR	8 weeks	Yes	Yes
Ouédraogo (2016), Burkina Faso^(27)^	26	13	11 months	Yes	Yes
Doris (2016), Ghana^(28)^	NR	NR	NR	Yes	Yes
Adamu (2016), Nigeria^(29)^	NR	NR	NR	Yes	Yes
Koroma (2020), Sierra Leone^(30)^	38	3	2 weeks	Yes	Yes
Kassa (2020), Ethiopia^(31)^	NR	NR	1 month	Yes	Yes

NR, not reported.

Information on the number of personnel employed and the length to complete the planned sample size was reported in eleven articles^(14,16,19–21,23–27,30)^, with an average study period of 22⋅5 d (range: 5–60 d). The number of interviewers ranged from ten (for a sample size of 900 units completed in 1 week) to fifty-three (for a sample size of 4480 units completed in 5 d). Interviewers were locally recruited and combined in teams of two, with each team completing an average of 29 HH/d. The number of supervisors varied widely. One study^(21)^ reported that gender balance had been ensured in each team to deal with local customs, while another study^(15)^ had recruited a statistician to support sample size estimation, sampling procedures and data analysis.

### Data analysis and outcome measured

Table 7 shows the data analysis procedures reported in the reviewed articles. Overall, eleven articles^(15,17,19,21–23,25,28–31)^ reported 95 % confidence intervals (95 % CI) for estimated VAS coverage, only four articles^(15,20,24,30)^ calculated weights for analysis to account for differences in population size within the sampled clusters and only three^(15,21,22)^ adjusted VAS coverage estimates for non-response rate. Most studies (*n* 13)^(15,20–31)^ reported VAS coverage by the age group (6–11 and 12–59 months) and two^(17,30)^ also by the collection method. Additionally, the CLQAS^(30)^ reported the number of lots that passed the defined VAS coverage threshold.

**Table 7.** Data analysis procedures and outcome measured by studies included in the systematic review of methodologies to measure the coverage of Vitamin A Supplementation (*n* 18)

Article	95 % CI reported	VAS coverage weighted for cluster sampling methodology	VAS coverage adjusted for non-response	VAS coverage presented by data source (card, recall)	VAS coverage presented by age group	Outcome measured	Month of study implementation
Bharmal (2001), Pakistan^(14)^	No	No	No	No	Yes	One-dose VAS coverage after an event	NA
Masanja (2006), Tanzania^(15)^	Yes	No	Yes	No	No	One-dose routine VAS coverage	NR
Bendech (2007), Guinea^(16)^	No	Yes	No	No	No	One-dose routine VAS coverage	June–August
Ayoya (2007), Mali^(17)^	Yes	No	No	Yes	No	One-dose VAS coverage after an event	NA
Sachdeva (2009), India^(18)^	No	No	No	No	No	One-dose routine VAS coverage	June–August
Gebremedhin (2009), Ethiopia^(19)^	Yes	No	No	No	No	One-dose routine VAS coverage	NR
Hodges (2013), Sierra Leone^(20)^	No	Yes	No	No	Yes	One-dose VAS coverage after an event	NA
Nyhus (2013), Tanzania^(21)^	Yes	No	Yes	No	Yes	One-dose VAS coverage after an event	NA
Olusegun (2013), Zambia^(22)^	Yes	No	Yes	No	Yes	One-dose routine VAS coverage	June–August
Hamadoun (2013), Mali^(23)^	Yes	No	No	No	Yes	Two-dose VAS coverage	December–February
Clohossey (2014), Kenya^(24)^	No	Yes	No	No	Yes	One-dose VAS coverage after an event	NA
Sesay (2015), Sierra Leone^(25)^	Yes	No	No	No	Yes	One-dose VAS coverage after an event	NA
Harsh (2015), India^(26)^	No	No	No	No	Yes	One-dose routine VAS coverage	June–August
Ouédraogo (2016), Burkina Faso^(27)^	No	No	No	No	Yes	Two-dose VAS coverage	NR
Doris (2016), Ghana^(28)^	Yes	No	No	No	Yes	Two-dose VAS coverage	December–February
Adamu (2016), Nigeria^(29)^	Yes	No	No	No	Yes	Two-dose VAS coverage	NR
Koroma (2020), Sierra Leone^(30)^	Yes	Yes	No	Yes	Yes	One-dose routine VAS coverage	September
Kassa (2020), Ethiopia^(31)^	Yes	No	No	No	Yes	One-dose routine VAS coverage	June

CI, confidence interval; NA, not applicable; NR, not reported; VAS, vitamin A supplementation.

Eight studies^(15,16,18,19,22,26,30,31)^ measured one-dose routine VAS coverage. Of these, six^(16,18,22,26,30,31)^ were carried out during the June–September period to estimate the proportion of children who received one vitamin A supplement in the first semester of the year.

Among studies measuring two-dose VAS coverage^(23,27–29)^, two^(23,28)^ were conducted during the December–February period to estimate the proportion of children who had received two vitamin A supplements in the previous year. In the LCS^(27)^, children who received a first dose of vitamin A in the first semester of the year were followed up in the second semester to assess whether they had received a second dose.

## Discussion

The systematic review revealed that across regions and time, CCS represented the principal method for measuring and validating VAS coverage, both after a vitamin A event distribution and via routine health contacts.

The majority of studies reviewed adapted the methodology of the WHO EPI cluster survey^(8)^, modifying sample size, sampling and data analysis procedures. In making such modifications, these studies partly implemented the new WHO recommendations contained in the 2018 Vaccination Coverage Cluster Surveys Reference Manual^(9)^.

In fact, over time, EPI surveys have increased in complexity, matching the evolution of the EPI since its inception in 1974 with the so-called ‘30 × 7 design’^(7)^.

Although the basic 30 × 7 EPI survey design has been a valuable programme management tool, the use of non-probability sampling and lack of standardised, well-documented quality control procedures may reduce confidence in the results^(32)^. To address these limitations, the WHO Vaccination CCS Reference Manual was updated in 2005^(8)^ and again in 2018^(9)^ and is still considered the standard guidance for conducting a CCS.

To calculate sample size, most studies used anticipated VAS coverage, a desired precision of ±5–10 %, a confidence level of 5 %, an average DEFF of 2⋅5 and a predefined number of clusters and respondents per cluster. In addition to these WHO-recommended parameters^(8,9)^, sample size should also be increased for an estimated non-response rate. However, this last parameter was only considered by four studies included in the systematic review^(26,28,29,31)^.

Rather than pre-establishing a certain number of clusters and HHs, the 2018 WHO Manual^(9)^ recommends that at least thirty clusters be selected per stratum of a minimum of ten respondents each. This was done in a majority of reviewed studies.

Most studies (*n* 15)^(15,16,18–26,28–31)^ also selected clusters with PPS that ensure representativeness, as larger units that represent a greater proportion of the population are more likely to be sampled^(9)^. Another advantage of PPS sampling is that it reduces variation among sampling weights, which reduces confidence interval width for coverage estimates.

Cluster segmentation was considered by five studies^(21,22,24,25,30)^; this technique is recommended to optimise resources when there are large clusters that have many more HHs than needed^(8,9)^.

Consistent with WHO recommendations^(9)^, seven studies^(16,19,20,22,24,25,31)^ defined clusters as EAs, which represent the smallest defined geographical units created for the enumeration purposes of the census and may already have maps and defined boundaries.

While most studies (*n* 12)^(15–24,26,28)^ employed the WHO random walk method for HH selection, this approach can introduce selection bias due to field worker decisions and practices^(32,33)^. The current recommendation^(9)^ is to randomly select HHs from a list of those within the selected cluster. This approach was used in two studies^(25,30)^.

When selected HHs are found empty or selected clusters become inaccessible (e.g. due to conflict, wildfires and flooding), a plan for cluster replacement and at least two HH revisits should be put in place. Yet overall, only one study^(16)^ reported having a replace/revisit plan.

Within the selected HHs, most studies enrolled only one eligible child aged 6–59 months. To optimise resources and guarantee that the probability of selection for an individual is equal to the probability of selection for his or her HH, WHO^(9)^ recommends including every eligible child in every selected HH, as was done in five reviewed articles^(22,23,27–29)^. Moreover, because the target population of VAS programmes is children aged 6–59 months, it is recommended that the whole age group be included to both optimise resources and measure the percentage of children 6–59 months of age who received an age-appropriate vitamin A supplement in each semester^(5)^.

Digital data collection is beneficial because it eliminates the problem of illegible handwriting and can be directly linked, via data transmission, to a central location for storage and analysis. It also makes it easier to check the entries for mistakes and correct them before the data are transmitted^(9)^. For these reasons, electronic tools, as used in only two reviewed studies^(28,30)^, are preferred over paper-based questionnaires, where feasible. In certain contexts, in fact, digital data collection may not be feasible due to lack of electricity, internet connection and capacity in addition to data security issues.

In all the articles included in the systematic review, the CHC and parent recall were the main sources of information on the number of vitamin A supplements received by the child. If no home-based record of VAS was available, the next level of evidence was a verbal history of VAS by parents. In immunisation surveys, the validity of parental recall can be unreliable because of the complexity of immunisation schedules^(34)^. However, remembering the number of vitamin A capsules received by the child is more straightforward and can be facilitated by showing a sample capsule to the parent, as was done in eleven studies^(15–17,19–22,24,27,30,31)^. For these reasons, it is acceptable to collect VAS information by parental recall when CHC is not available.

According to WHO^(8,9)^, to guarantee the quality of collected data, it is necessary to provide interviewers with training and supervision. They should be organised in teams of two completing one cluster of a maximum of 30 HH/day. One supervisor should also be assigned to every two teams to monitor the quality of their work. WHO also recommends that interviewers be familiar with the clusters they are assigned and fluent in the local language. In line with these recommendations, most studies (*n* 14)^(14–21,23–25,27,30,31)^ provided training and supervision to locally recruited interviewers, who surveyed an average of 29 HH/d.

Once data are collected, recommended quality actions^(8,9)^ include double data checking, entry and cleaning. These actions were performed in the majority of reviewed studies (*n* 12)^(14–16,19–22,24,25,27,29,31)^.

Subsequently, under the multistage cluster sampling approach with PPS, data analysis must be weighted because sampling probabilities differ for different respondents. To derive a correct coverage estimate, sample weights need to be applied to each cluster to account for differences in population size and for non-response^(8,9)^. Overall, only four studies^(16,20,24,30)^ calculated weights for analysis to account for differences in population size within the sampled clusters, and another three studies^(15,21,22)^ adjusted VAS coverage estimates for non-response rate. On the other hand, the majority of studies (*n* 11)^(15,17,19,21–23,25,28–31)^ reported a 95 % CI of estimated VAS coverage, as recommended by WHO^(8,9)^, including the CLQAS^(30)^ which design is not meant to measure the point of coverage estimates, but to identify whether an area (lot) has achieved a minimum level of coverage^(32)^. Although the main outcome of CLQAS is a binary classification of areas (lots) in accepted/rejected, without providing a point of coverage estimate, lot data can be aggregated according to a stratified weighted design to estimate coverage in the area. The main advantage of CLQAS is the small sample size required to classify lots with regard to coverage levels, but despite such advantage, the only reviewed CLQAS^(30)^ selected 855 units, no more no less of the CCSs. Moreover, WHO discourages the use of this design to measure the point of coverage estimates, as it is not specifically conceived for this goal and uses *a priori* defined decision rules to classify coverage which contrast with the objective of coverage estimation^(9)^.

Because implementing a coverage survey is resource-intensive, efforts should be made to improve efficiency by measuring annual two-dose VAS coverage, presenting data by the age group (i.e. 6–11 and 12–59 months) and by the collection method. To do this, the survey should be performed during the December–February period, as done by two of the reviewed studies^(23,28)^.

Following-up with children to assess if they receive their second dose, as done in the LCS^(29)^, may introduce selection biases (e.g. by not considering population movement, including newly arrived children and children who age in or out of the eligible age range between the first and second dose). WHO underlines that an important sampling challenge is ensuring that no populations are missed, especially those that are difficult to reach^(32)^.

In accordance with ethical standards^(35)^, most studies (*n* 15)^(14–16,19–24,26–31)^ were conducted in accordance with national policies on ethics for surveys involving human subjects, including obtaining verbal informed consent, which is widely accepted by Institutional Review Boards for a standard coverage survey without biological sample collection^(9)^.

It is a limitation of this systematic review that six studies^(14,16,18,20,24,27)^ did not provide information on sample size calculation procedures. Moreover, only five articles^(19,21,25,29,30)^ were classified as being of high quality.

The review is also limited by the small number of studies focusing on the measurement of routine VAS coverage (*n* 8)^(15,16,18,19,22,26,30,31)^ and, in particular, two-dose routine VAS coverage (*n* 4)^(23,27–29)^.

While a greater number of studies would have provided a wider evidence base upon which to draw conclusions, the lack of peer-reviewed publications itself demonstrates the need to strengthen methods for measuring the administrative coverage of VAS delivered through routine health services.

## Conclusion and recommendations

In the current transition process towards routine health system contacts as the main VAS delivery platform and administrative electronic-based data collection systems, improving routine data quality is the best way to ensure stronger service delivery and monitoring of VAS programmes, as these data provide the most sustainable method for coverage estimation.

However, most VAS priority countries are in the early stages of this process and do not yet have the ability and full capacity to measure routine two-dose VAS coverage^(4)^.

Based on the results of this systematic review, these countries can adopt multistage CCS to measure VAS coverage, using the recommendations included in Table 8.

**Table 8.** Recommendations to conduct vitamin A supplementation coverage cluster survey

**Pillars** Measure two-dose vitamin A supplementation (VAS) coverage performing the coverage cluster survey during December–February Include the whole age group of children aged 6–59 months
**Sample size calculation** Use anticipated VAS coverage from previous surveys conducted in the study area. If not available, UNICEF provides country estimates (https://data.unicef.org/topic/nutrition/vitamin-a-deficiency/) Define desired precision between ±5 and 10 % Define a confidence level at 5 % Define a design effect of ≥2⋅4 Predefine at least thirty clusters per stratum, each of a minimum of ten respondents Increase the sample size for an estimated non-response rate; if not available use the value of 7⋅5 %
**Sampling procedures** When available, define clusters as census enumeration areas, or similar Select clusters with probability proportional to size Use cluster segmentation technique, if needed Avoid using the random walk method; instead randomly select households from a full list Enrol every eligible respondent in every selected household
**Data collection** Consider digital data collection tools Collect information from the child health card; if a card is not available, collect from parental recall and showing a sample of VAS capsules
**Data quality assurance** Pre-test and translate data collection tools in the local language Provide training to interviewers and ensure field supervision Recruit interviewers that are familiar with the clusters they are assigned to and fluent in the local language Ensure double data checking, entry and cleaning Include a plan for households and clusters revisit and replacement
**Planning considerations** Divide interviewers into teams of two completing one cluster of a maximum of thirty households per day. Consider gender balance to deal with local customs, if needed Ensure a maximum of one supervisor for every two teams Consider recruiting an expert statistician Consider an average of 22⋅5 d for fieldwork Obtain ethical clearance Request verbal informed consent from caregivers
**Data analysis** Calculate weighted VAS coverage accounting for differences in population size within the sampled clusters and for non-response rate Report 95 % CI of estimated VAS coverage Report one-dose and two-dose VAS coverage by the target age group (6–11 and 12–59 months) Report VAS coverage by the collection method (card, recall and card plus recall)

Consistent with WHO guidance^(9)^, the methodological recommendations provided will enable and support countries to collect reliable data for VAS coverage measurement (either after a vitamin A event distribution or via routine health contacts) in order to plan, monitor and evaluate VAS programmes in the current transition period and beyond.

## Supplementary material

For supplementary material accompanying this paper visit http://dx.doi.org/10.1017/jns.2021.65. click here to view supplementary material

## References

[1] Imdad A, Mayo-Wilson E, Herzer K, (2017) Vitamin A supplementation for preventing disease and death in children aged six months to five years. Cochrane Database Syst Rev 3, 3–18. 10.1002/14651858.CD008524.pub3 PMC6464706 28282701

[2] World Health Organization (2011) Vitamin A Supplementation for Infants and Children 6–59 Months of Age. Geneva: World Health Organization. http://www.who.int/nutrition/publications/micronutrients/guidelines/vas_6to59_months/en/ (accessed 27 May 2020).

[3] World Health Organization (2021) Micronutrient Deficiencies: Vitamin A Deficiency. Geneva: World Health Organization, https://www.who.int/nutrition/topics/vad/en/ (accessed 27 May 2020).

[4] United Nations Children's Fund (2018) Coverage at a Crossroads: New Directions for Vitamin A Supplementation Programmes. New York: United Nations Children's Fund. https://www.unicef.org/publications/index_102820.html (accessed 27 May 2020).

[5] United Nations Children's Fund (2020) Estimates of Vitamin A Supplementation Coverage in Preschool-Age Children: Methods and Processes for the UNICEF Global Database. New York: United Nations Children's Fund. https://data.unicef.org/resources/vitamin-a-coverage-methodology-2020/ (accessed 27 May 2020).

[6] Janmohamed A & Doledec D (2017) Comparison of administrative and survey data for estimating vitamin A supplementation and deworming coverage of children under five years of age in sub-Saharan Africa. Trop Med Int Health 22, 822–829. 28449319 10.1111/tmi.12883

[7] The Global Alliance for Vitamin A (GAVA) (2014) *A Guide For Conducting Post*-*Event Coverage Surveys* f*or Vitamin A Supplementation, Deworming* a*nd Immunization Events*. Gava: The Global Alliance for Vitamin A. http://www.gava.org/content/user_files/2016/12/PECS-Manual-V2-3-12-14-English.pdf (accessed 27 May 2020).

[8] World Health Organization (2005) Immunization Coverage Cluster Survey – Reference Manual. Geneva: World Health Organization. http://whqlibdoc.who.int/hq/2005/WHO_IVB_04.23.pdf (accessed 27 May 2020).

[9] World Health Organization (2018) World Health Organization Vaccination Coverage Cluster Surveys: Reference Manual. Geneva: World Health Organization. http://apps.who.int/iris/bitstream/handle/10665/272820/WHO-IVB-18.09-eng.pdf?ua=1 (accessed 27 May 2020).

[10] Corsi DJ, Neuman M, Finlay JE, (2021) Demographic and health surveys: a profile. Int J Epidemiol 41, 1602–1613. 10.1093/ije/dys184 23148108

[11] Multiple Indicator Cluster Surveys (2020) United Nations Children's Fund. http://mics.unicef.org/tools (accessed 27 May 2020).

[12] Moher D, Liberati A, Tetzlaff J, (2009) Preferred reporting items for systematic reviews and meta-analyses: the PRISMA statement. PLoS Med 6, 6–17. 10.1371/journal.pmed.1000097 PMC2707599 19621072

[13] Modesti PA, Reboldi G, Cappuccio FP, (2016) Newcastle–Ottawa quality assessment scale (adapted for cross sectional studies). PLoS One 11, 1.

[14] Bharmal FY & Omair A (2001) Evaluation of vitamin A supplementation in Gulshan-e-Sikandarabad. J Pak Med Assoc 51, 248–250. 11558216

[15] Masanja H, Schellenberg JA, Mshinda HM, (2006) Vitamin A supplementation in Tanzania: the impact of a change in programmatic delivery strategy on coverage. BMC Health Serv Res 6, 142. 17078872 10.1186/1472-6963-6-142 PMC1635705

[16] Bendech MA, Cusack G, Konaté F, (2007) National vitamin A supplementation coverage survey among 6-59 months old children in Guinea (West Africa). J Trop Pediatr 53, 190–196. 17463012 10.1093/tropej/fmm007

[17] Ayoya MA, Bendech MA, Baker SK, (2007) Determinants of high vitamin A supplementation coverage among pre-school children in Mali: the National Nutrition Weeks experience. Public Health Nutr. doi:10.1017/S1368980007687138. 17381941

[18] Sachdeva S & Datta U (2009) Vitamin A-first dose supplement coverage evaluation amongst children aged 12–23 months residing in slums of Delhi, India. Indian J Ophthalmol 57, 299–303. 19574699 10.4103/0301-4738.53056 PMC2712700

[19] Gebremedhin S, Loha E, Abebe Y, (2009) Assessment of vitamin A supplementation coverage and its association with childhood illness in Boloso Sore Woreda, Welayta Zone, SNNP Region, Ethiopia. Ethiop J Health Dev 23, 223–228.

[20] Hodges MH, Sesay FF, Kamara HI, (2013) High and equitable mass vitamin A supplementation coverage in Sierra Leone: a post-event coverage survey. Glob Health Sci Pract 2, 172–179. 10.9745/GHSP-D-12-00005 PMC4168566 25276530

[21] Dhillon C N, Subramaniam H, Mulokozi G, (2013) Overestimation of vitamin a supplementation coverage from district tally sheets demonstrates importance of population-based surveys for program improvement: lessons from Tanzania. PLoS One 8, e58629, doi:10.1371/journal.pone.0058629. 23536804 PMC3594174

[22] Babaniyi O, Siziya S, Mukonka V, (2013) Child Nutrition and Health campaign in 2012 in Zambia: coverage rates for measles, Oral Polio Vaccine, Vitamin A, and de-worming. Open Vaccine J 6, 1–8.

[23] Sangho H, Belemou B, Keita HD, (2013) Processus de supplémentation en vitamine A chez les enfants de moins de cinq ans lors d'une semaine d'intensification des activités de nutrition au Mali (Vitamin A supplementation in children under five during a one-week nutrition intensification program in Mali). Sante Publique 25, 821–827. 24451428

[24] Clohossey PC, Katcher HI, Mogonchi GO, (2014) Coverage of vitamin A supplementation and deworming during Malezi Bora in Kenya. J Epidemiol Glob Health 4, 169–176. 25107652 10.1016/j.jegh.2013.12.005 PMC7333821

[25] Sesay FF, Hodges MH, Kamara HI, (2015) High coverage of vitamin A supplementation and measles vaccination during an integrated Maternal and Child Health Week in Sierra Leone. Int Health 7, 26–31. 25316706 10.1093/inthealth/ihu073

[26] Mahajan H, Srivastav S & Mukherjee S (2016) Coverage of vitamin A supplementation among under-five children in an urban resettlement colony of district Gautam-Budh Nagar, Uttar Pradesh. Int J Med Sci Public Health 5, 1328–1333.

[27] Ouédraogo CT, Becquey E, Wilson SE, (2016) Factors affecting the validity of coverage survey reports of receipt of Vitamin A supplements during child health days in southwestern Burkina Faso. Food Nutr Bull 37, 529–543. 27604622 10.1177/0379572116666167

[28] Hadzi D, Asalu GA, Avedzi HM, (2016) Vitamin A supplementation coverage and correlates of uptake among children 6–59 months in the South Dayi District, Ghana. Cent Afr J Public Health 2, 89–98.

[29] Adamu MD & Muhammad N (2016) Assessment of Vitamin A supplementation coverage and associated barriers in Sokoto State, Nigeria. Ann Nigerian Med 10, 16–23.

[30] Koroma AS, Conteh SG, Bah M, (2020) Routine vitamin A supplementation and other high impact interventions in Sierra Leone. Matern Child Nutr 16, e13041. 32720469 10.1111/mcn.13041 PMC7507363

[31] Kassa G, Mesfin A & Gebremedhin S (2020) Uptake of routine vitamin A supplementation for children in Humbo district, southern Ethiopia: community-based cross-sectional study. BMC Public Health 20, 1500. 33008352 10.1186/s12889-020-09617-1 PMC7532605

[32] Danovaro-Holliday MC, Dansereau E, Rhoda DA, (2017) Collecting and using reliable vaccination coverage survey estimates: summary and recommendations from the ‘Meeting to share lessons learnt from the roll-out of the updated WHO Vaccination Coverage Cluster Survey Reference Manual and to set an operational research agenda around vaccination coverage surveys’, Geneva, 18–21 April 2017. Vaccine 36, 5150–5159. 10.1016/j.vaccine.2018.07.019 PMC6099121 30041880

[33] Grais RF, Rose AM & Guthmann JP (2007) Don't spin the pen: two alternative methods for second-stage sampling in urban cluster surveys. Emerg Themes Epidemiol 4, 8. doi:10.1186/1742-7622-4-8. 17543102 PMC1894792

[34] Miles M, Ryman TK, Dietz V, (2013) Validity of vaccination cards and parental recall to estimate vaccination coverage: a systematic review of the literature. Vaccine 21, 1560–1568. 10.1016/j.vaccine.2012.10.089 23196207

[35] World Health Organization (2011) Standards and Operational Guidance for Ethics Review of Health-Related Research with Human Participants. Geneva: World Health Organization. https://apps.who.int/iris/bitstream/handle/10665/44783/9789241502948_eng.pdf;jsessionid=1C780EDDFF80D9CFA0A42DF0E25DCD70?sequence=1 (accessed 27 May 2020). 26269877

