# Supplementary material for: Methodologies to measure the coverage of vitamin A supplementation: a systematic review
Source: J Nutr Sci. 2021 Aug 27;10:e68. doi: 10.1017/jns.2021.65 (PMC8411257; doi:10.1017/jns.2021.65)
Supplement: Supplementary file 1 [file S2048679021000653sup001.docx]

**Supplementary material 1.** Glossary of terms used in the systematic review of methodologies to measure Vitamin A Supplementation Coverage.

| **Administrative vitamin A supplementation (VAS) coverage:** Monthly tally sheet, paper or electronic register, which records each month the VAS doses administered at a health post or other routine health system platforms. Recorded doses are then aggregated upward to district, region and national level to provide a total numerator (i.e. total number of children who received a dose). Monthly data is then aggregated for the first six months of the year, to estimate the total number of children reached in the first semester. This is repeated for the second semester, with the intention of being able to estimate annual two-dose VAS coverage.  **Anticipated vitamin A supplementation coverage**: This value can be taken from previous surveys conducted in the country or study-area. The value is then used in the formula for sample size estimation.  **Cluster:** A collection of elements (for example, households, communities, villages, census enumeration areas, etc.) grouped within defined geographical or administrative boundaries.  **Clusters segmentation:** Technique used to divide clusters into geographical segments to be randomly selected. Clusters could be divided into segments that are estimated to have (a) at least the target number of households per cluster and (b) no more than two times the target number of households per cluster.  **Cluster coverage survey (CCS):** A survey in which the population under study is divided into an exhaustive and mutually exclusive set of primary sampling units (clusters), and a subset of those clusters is randomly selected for sampling.  **Confidence interval (CI):** A range or interval of parameter values around the VAS coverage point of estimate that is meant to be likely to contain the true population parameter.  **Confidence level**: A level of confidence set when computing confidence limits. A level of 95% (or 0.95) is conventionally used, and implies that 19 out of 20 times the VAS coverage from a survey using this level will capture the true population value.  **Confidence limits:** The upper and lower limits of a confidence interval. The interval itself is called the confidence interval or confidence range. Confidence limits are so called because they are determined in accordance with a specified or conventional level of confidence or probability that these limits will, in fact, include the population parameter being estimated. Thus, 95% confidence limits are values between which we are 95% confident that the population parameter being estimated will lie.  **Convenience sampling:** Selection of sampling units (households, clusters etc.) by convenience (i.e. proximity, accessibility, etc.).  **Design effect (DEFF):** A measure of variability due to selecting survey subjects by any method other than simple random sampling. It is defined as the ratio of the variance with the chosen type of sampling to the variance that would have been achieved with the same sample size and simple random sampling. Usually, cluster surveys have a design effect greater than one, meaning the variability is higher than for simple random sampling.  **Desired precision:** The level of absolute precision specifies the width of the confidence interval. For example, if the desired precision is set at 0.5 and the VAS coverage is 80%, the 95% CI will range around the VAS coverage point estimate of ±5% (i.e. 75%-85%).  **Expanded Programme on Immunization (EPI) cluster survey methodology**: A survey done in 30 systematically selected clusters of seven or more children to estimate the coverage for a health intervention among all the children that live in the area (i.e. the population) being surveyed.  **Household (HH):** A group of persons who live and eat together, sharing the same cooking space/kitchen.  **Longitudinal Cluster Survey (LCS)**: A survey where data in each variable is measured repeatedly over time. One way of analyzing this type of data is to cluster them; i.e., divide the population into homogeneous subgroups.  **Lot quality assurance sampling (LQAS) Survey:** A survey design to classify areas of interest (lots) as having reached acceptable or unacceptable levels of coverage of a health intervention (e.g. immunizations, VAS, etc.). LQAS uses two coverage thresholds, an upper threshold (UT) and a lower threshold (LT), and a decision value (d) in a sample (N) of the population. If the number of individuals who have not received the health intervention found in N is higher than d, then the lot is rejected as not having reached an acceptable coverage (i.e. real coverage in the lot is below UT); if it is equal to or less than d, then the lot has an acceptable coverage (i.e. real coverage in the lot is above LT).  **Multi stage cluster sampling:** A survey design with more than one stage of selection to identify the respondents to be interviewed. This might involve randomly selecting clusters and then randomly selecting households. It might also involve stratifying the sample and conducting a survey in each stratum, using one or more sampling stages.  **Probability proportional to size (PPS):** An unequal probability sampling technique used for cluster selection, in which the probability of selection for each sampling unit is proportional to its size (i.e. number of households, number of eligible, etc.).  **Random selection:** A sample drawn from a set of eligible units or participants where each unit or participant has an equal probability of being selected.  **Response rates**: A measure that take into account the potential response or compliance of the population to be surveyed. The value can be taken from previous surveys conducted in the country or study-area.  **Stratum (plural: strata):** A stratum is a portion of the population for which survey results will be estimated and reported with a desired level of precision on the survey's primary outcome. It could be defined as urban/rural areas, or according to Country’ administrative division (i.e. Districts, Provinces, Regions, etc.). Within a stratum a number of clusters are sampled.  **Weight:** A number that expresses the relative number of population units represented by a given sampled unit. It indicates how much each record or case will count in a statistical procedure. Each record in a survey dataset might be accompanied by one or more survey weights, to indicate how many population level eligible respondents are represented by the respondent in the sample. The design weight is the reciprocal of the probability that the respondent was selected to participate in the survey, which is the product of the probabilities of selection at each stage.  **World Health Organization (WHO) random walk method:** The random walk method is a simplified household selection method developed by the WHO and originally implemented in the Expanded Programme on Immunization (EPI). It consists in spinning a bottle from the centre of a sampled cluster to determine a direction to follow. The households along that direction are then visited until the number of determined children to be enrolled is reached. Several “modified” random walk methods have been proposed which allows to reduce selection bias, and mainly consists in spinning a bottle from the centre of a sampled cluster to determine a direction to follow, count all the households in the selected direction out to the boundary of the cluster, assign a number to the counted households randomly selecting the households to visit. |
| --- |
